# Treatment of Pulpless Teeth

**Published:** 1885-09

**Authors:** H. A. Knight

**Affiliations:** Minneapolis


### Treatment of Pulpless Teeth.

BY H. A. KNIGHT, D.D.S., MINNEAPOLIS.

Read at the Minnesota State Dental Society.

In this paper I shall take the liberty to include under the head of pulpless teeth all which are so diseased as to require devitilization of the pulp, and subsequent treatment, and divide my paper into three sections, namely : Diagnosis, prognosis, and treatment.

Diagnosis, coming from the words dia, through, and gnosis, knowledge, means in medicine to be able by our knowledge of symptoms to name the disease, and upon our diagnosis depends our prognosis and treatment. Can he who makes no diagnosis intelligently treat disease, or can he expect a favorable termination of his treatment? Must he not rather blindly go forward with his treatment, after some stereotyped plan, earnestly praying for success, and like so many physicians, more fit to do farm work than practice medicine, meet almost constantly with failure.

Not many years ago an aching tooth was fit only for the forceps, and the dentist, if I may call him such, only asked a few questions before applying the remedy. The sacrifice of a tooth was not a sacrifice in the patient's eyes, but a blessing, for people had been taught from time immemorial, that nothing could be done for a tooth after it had begun to trouble. An aching tooth to the dentist was an aching tooth; he knew no difference between pulpitis, periostitis, and alveolar abscess. They were all the same to him, and required the same treatment. Of diagnosis he knew nothing, of medicine less, but of prognosis he had a clear understanding, for it was always the same. If a tooth had never given signs of trouble, and the cavity was the size of a pin head, favorably located, he managed to excavate and fill it, oftentimes very acceptably, while the badly decayed teeth and roots were removed and replaced by beautiful white artificial teeth.

The dentist of those days was but a mere mechanic, and what mattered it if the dentist cut hair and shaved you when occasion required. I speak of the times gone by, but they have not gone by. It was but recently that a prominent dentist of our sister city asked me what I treated teeth with, and I heard not long ago of a member of our local society say that he used carbolic acid for everything in his office and that it was the best remedy he had ever found. Imagine that dentist applying carbolic acid to every case in practice, and then wonder if you can, that he finds teeth that are incurable.

Diagnosis of every case must be made, in order to intelligently apply the remedies within our reach. A case of congestion of the pulp must no more be treated for periostitis than alveolar abscess for necrosis.

We, as intelligent dentists, should be able to clearly define and comprehend the case in hand before we even dare to turn to our cabinet for a remedy. With the common symptoms of dead teeth, such as occasional slight pain, tenderness of pressure, discoloration, elongation, looseness and congestion of the surrounding parts, we are all familiar, but I bring to mind a class of teeth with dying and sloughing pulps, complicated with slight periostitis whose symptoms so nearly resemble those of a congested pulp as to make a clear diagnosis almost an impossibility. Up to this period you have no marked symptoms of periostial disturbance, and the gum gives no evidence of the serious trouble brewing beneath its surface.

Take for instance a typical case in practice: The patient comes in suffering with toothache in the upper first bicuspid, which may have a large proximal cavity in it, or perchance a filling but lately inserted. All the patient knows of the case is that thermal changes affect it and the pain is most severe. The first question arising in your mind is, what condition is that pulp in? is it safe to take the engine and bore directly into the pulp cavity? In a simple congestion, as well as pulpitis, the tooth is affected by heat and cold, and may be slightly sensitive on percussion; the surroundings remain in an apparently healthy condition, the tooth is firm in the socket, and no cause for the severe pain, is visible externally. Just when the congestive period has passed, and the inflammatory process been set up, is a difficult problem to solve, but much dependence may be placed on a minute history of the case. When questioned closely, the patient will almost invariably tell you that the first pain experienced was caused by some cold substance coming in contact with the tooth; that the pain was only of short duration, but that as time passed, the trouble occurred at more frequent intervals and upon slighter provocation; even the ordinary atmosphere causing intense suffering. Up to this period, anything warm coming in contact with the tooth, affords relief, but from this time forward affairs seemed to be reversed. Heat becomes the troublesome agent and cold the pacifier, while the pain, instead of making short calls during the day, occurs almost without fail at night, and usually prolongs its visits several hours. It is at this time the periosteum begins to show disturbance, and the pulp passes into dissolution ; the tooth becomes elongated and painful upon the slightest pressure.

When a tooth reaches this stage I class it with pulpless teeth, for to my mind, the pulp has lost its chance for resolution to take place, and if not destroyed by some external agent will in spite of all remedy die.

We have coming before our notice all forms of so-called dead teeth, some with pulps dead and putrescent, but intact, some having lost all trace of a pulp, and others, where the disease has progressed far enough to cause alveolar-abscess, or necrosis of the jaw. The diagnosis of these latter varieties is not a very difficult matter. By tapping a tooth known to be dead, periostitis, be it ever so slight, can usually be determined. An abscess may be diagnosed by the looseness which usually accompanies it by the turgid appearance of the gum and by fluctuation when the pus has pierced the bone and formed in sufficient quantity. Where necrosis of the jaw is found to accompany an abscess, it may be distinguished by an odor peculiar to the pus of carious bone in any other part of the body.

After thoroughly understanding the pathological condition attendant upon the various forms of diseased and pulpless teeth, a minute history of each case should enable any dentist to begin treatment intelligently, and feel warranted in making a favorable prognosis.

In a vast majority of cases, where a tooth can be properly treated and filled, I think the dentist is warranted in assuring the patient of ultimate success, but at the same time many things must be considered in the treatment. Some constitutional diseases, although exhibiting themselves in more marked form on some other parts of the body, exert a special influence upon the teeth and associate parts and must not be overlooked any more than the diathesis of a patient. It would be folly for any dentist to think that a conquest could be made as easily in the case of a patient with an inflammatory diathesis exhibited by plethore and increased capillary activity, accompanied perhaps by the habitual use of alcoholic beverages and stimulating food, as in the case of a young vigorous individual with a clear constitution. Experience has taught us that that class of people, as well as the pale anæmic individual, whose every look indicates perverted nutrition, and the elderly person with enfeebled circulation and nerve force present many very obstinate complications.

The influence of an inflammatory diathesis in the treatment of teeth may be very great, as is often exhibited by the scratch of a pin. I have met people upon whom the slightest bruise seemed sufficient cause for intense inflammation and sloughing, requiring weeks for a complete cure. In such a case, healing by first intention is an impossibility. The blood does not seem to possess reparative material enough even for the natural waste, to say nothing of the accidental. The system, instead of building up, seems inclined to go into dissolution. The influence of malaria upon this class of teeth is often noticed, as well as in the treatment of exposed pulp, and tends to excite the parts when we first begin treatment. According to one writer, it may be detected by the serum or watery part of the blood oozing up through the root. Add to the above causes of modification the effect of gout, rheumatism, scrofula, and other cachexia, upon the human system, and the treatment of dead teeth becomes a profound study, each case presenting peculiarities which must not be overlooked.

Our prognosis must only be made after a careful study of the case, and if any doubt exists as to the outcome, we must have the benefit.

The treatment of pulpless teeth is a subject broad in area and full of rich material for thought, for no portion of dentistry has met with more discussion and experiment by the great mass of thinking dentists than this.

Within the memory of some of us, pulpless roots were considered foreign bodies buried in the jaw, of which nature constantly tried to rid herself. The healthy retention of these roots never occurred to the dentist or seemed possible, but the time has come when a dentist who cannot save a very large per cent. of them is considered many years behind in the knowledge of his profession.

The teeth are susceptible of treatment the same as any other part of the human organism. If we have an inflammation in or around a tooth, we have an irritant also. There is always a cause for an effect, and before we begin treating a case we should understand the conditions thoroughly. In order to successfully treat the teeth we must first determine in our minds just what we wish to do and the best method of accomplishing our end, selecting our remedy for a distinct purpose. It is only in this way we can expect the best results, and it is for lack of this so many failures are recorded.

In filling pulpless teeth, a few requirements must be met. The surrounding of the tooth must be free from disease or irritation, the root must be firm in the socket, the canal well excavated, all putrefactive processes arrested, and the canal well filled to the apex of the root. In order to meet these various requirements we must have full and free access to all parts of the tooth, and here let me say: Don't spare the tooth substance for the sake of appearance. I do not maintain that you must excavate each root to the end, for that would be impossible, but the nearer the apex of each root you get, and the more thoroughly you cleanse and fill the same, the better will be your chance of success.

A few days ago a case came under my notice where a dentist met with a great deal of trouble with a central incisor, simply because he could not get at the pulp.

A dentist of Freeport, Indiana, attempted to fill a small approximal cavity in a central incisor with gold. Some few days after the operation the thermal change affected the tooth seriously enough to cause him to remove the filling and replace it with a phosphate. This in turn was removed, and a filling of Hill's stopping substituted, but to no effect; the tooth still troubled, and the doctor resolved upon the destruction of the pulp, which he accomplished by access gained through this cavity, and applying his acid at the coronal extremity. After death of the pulp he attempted to remove it through this same cavity without enlarging the opening, which was scarcely larger than the broach itself. He simply attempted an impossibility, and punched on this semi-sensitive mass, time after time, with the result of packing the pulp into the upper half of the canal. This operation was repeatly followed by hemorrhage, but at the end of a month he pronounced it ready for filling, and made an engagement. The patient removed here before the day set, and fell into my hands. I mistrusted affairs were not all right, and so opened up from the palatine surface and removed the pulp. What result but an alveolar abscess could he have looked for had he finished his operation? As a usual thing, when you destroy a tooth pulp, you have no serious complications; the only part interfered with to a great extent, is the pulp, which must be removed to the apex. Ordinarily, it does not become necessary to apply any medical agent, simply syringing out the canal with tepid water being all that is required.

If no hemorrhage follows the removal of the pulp, you need not fear immediate filling, but in case of hemorrhage, it is safer to wait twenty-four or forty-eight hours, then syringe and fill.

In the treatment of pulpless teeth, no greater mistake has ever been made than over-treatment. This is a mistake the best of us are very prone to make, and although we may use the proper remedy, success will come only with its intelligent application. Nature is one of the most powerful of all healers, and if the surroundings of a tooth are given a fair chance, after removing the cause of irritant, in a great majority of cases they will return to their physiological condition.

An alveolar abscess may be said to be a discharging sore, within the aveolus, the result of inflammation and suppuration of the immediate parts. Its origin may usually be found in some local irritant, which causes a contraction of the blood-vessels, followed by an equal distention. The result is an increased flow of blood to the part or congestion and severe pain. If this congestion can in some way be relieved at this time, resolution will take place, but if it is allowed to proceed, complete stagnation will be the result, followed by suppuration and a discharge of pus.

To relieve the congestion preceding an abscess, many agents have been employed. The object sought is to change the blood pressure from the head to some more remote part of the body. After removing all the irritants or causes of irritation, a good liberal dose of physic is found very beneficial, in connection with a hot mustard foot bath, the feet and limbs being rubbed into a glow. Counter-irritation also is very helpful, and the application of tincture of aconite and iodine, equal parts, directly to the gum, has been useful in my experience. Iodine alone is also very good, painted on the gum after wiping the mucus away. Poultices made of capsicum, or capsicum and zingiber, are perhaps as capable of giving relief as any of the various forms of counter-irritants.

In relieving congestion and preventing an abscess forming, prompt treatment must be the rule, for after a certain time has elapsed it seems almost impossible for resolution to take place, and when we have forfeited this chance, the quicker an abscess forms the better. The length of time intervening between inflammation and suppuration may vary from a few hours to weeks and even months, according to the severity of the irritation and the physiological condition of the patient, and I have found in my experience the length of time required to cure an acute abscess is in proportion to the time it was in forming. The treatment of an abscess must always depend on the conditions existing when it comes under our notice. An acute abscess, the result of pent up gases or irritation, caused by working on a dead root or by accident, does not need any very great amount of care. If nature is assisted in relieving herself of the irritating pus formed, and thorough drainage supplied, with the root properly filled, in a majority of cases a speedy cure will be the result; but if the abscess has for some time been laboring at a disadvantage, and although less severe than formerly, taken in that chronic condition characterized by thin watery pus, the use of strong drugs will be more urgently demanded. We must first remove the prime irritant, which is usually a decomposed pulp, then supply the drainage for the purulent pus and endeavor to change the surface of the abscess from a passive state of inflammation to the acute. By proper stimulation the surface must be made to produce healthy granular new formation, instead of irritating pus. We must employ some strong drug or escharotic that will change the character of the secretion formed and be a sufficient irritant to excite healthy action. Oftentimes one good thorough application of carbolic or sulphuric acid will so change the character of an abscess as to enable nature to complete the cure. This may be accomplished through the foramen of the root, and in case of a fistulous opening, should be applied until it makes its exit at the gum. Many other agents have been recommended in the place of these, among which are worthy of mention, salycilic acid, iodoform, extract of eucalyptus, etc.

A very good remedy I find in creosote and iodine combined, as much iodine being put in as the creosote will dissolve. This makes a very penetrating volatile oil, which if left in the pulp canal even, will find its way to all parts of the cavity of disease. This combination possesses very nearly all the requisites in treatment: it is a disinfectant, antiseptic, and an irritant.

The class of teeth most difficult of treatment are those having a chronic blind abscess. The first thing to be done in these cases is to secure an opening through the gum, and here is afforded an opportunity for the believers in heroic treatment to demonstrate the success of their theory. If a tooth in this condition is excavated and filled, the gases forming will cause great pain and distention of the alveolus, and the result will be piercing of the bony walls and an opening in the gum. This condition of affairs, it seems, must be arrived at before thorough treatment can be made, and here the heroic dentist asks, "Is it not better to secure this opening by the use of cocaine and the burr, than to cause all this suffering?"

Another method of treating all forms of abscessed teeth has been to replant them; you simply extract the tooth, fill perfectly and return to its former position. Numerous cases are on record where this treatment has been very successful, but the absorption of the root of a replaced tooth is very likely to occur and let the tooth drop out like a temporary tooth.

The filling of the root of a dead tooth is perhaps as important a factor in successful treatment as any. There is simply one great law to be observed, namely: Filling the root perfectly with some indestructible material. It is easy enough to say all roots must be perfectly filled to the apex, but not so easy a thing to do. In some cases it seems almost impossible to follow the tortuous canals, even with a fine broach for the purpose of cleansing them, and we find sometimes the openings so small as to not even admit a broach. Numerous devices have been invented, from time to time, for enlarging these canals, and some of them work very nicely. Dr. Talbot, of Chicago, invented a system of reamers, with which no doubt you are all familiar. They consist of a number of triangular pyramidal points of various sizes, bent at different angles, intended for hand use. By rotation they enlarge the canal, removing the chips upon withdrawal, but it has been found impossible to follow a canal with them, and they have fallen slightly into disuse. A very neat little modification of them has been made lately, which I will exhibit. It overcomes the objection of not following the canal, and I have found it very useful.

The Gates Glyddon Drill has been found very convenient by some, but I have had bad luck with it and discarded it.

In reaming out a root, we should be cautious never to puncture the side of the root; if it is impossible to follow the canal, it is better to stop right where you are. Your canal having been prepared, the next thing is a choice of filling material; the case must determine the selection. The utility of each has been amply demonstrated, whalebone, wood, lead, gold, zinc, gutta-percha, and in fact, almost everything has been used. The object to be sought is to make a perfect filling, and what might be successful in one dentist's hands might prove a failure in another's.

The most difficult roots to fill are the buccal roots of upper molars. Here some plastic material must be used, and gutta-percha cut in chloroform, or zinc phosphate mixed to the consistency of cream may be made to answer the purpose.

The filling of the canals of the anterior ten teeth, is easily accomplished and the material used is a mere matter of choice with the dentist.

In the treatment of pulpless teeth it is well to observe a few rules:

1. Diagnose your case.
2. Select each remedy for a distinct purpose.
3. Avoid over-treatment.
4. Do not spare tooth substance at the expense of sight.
5. Fill as perfectly as possible each root at its apex.

